# Multi-adapter SAM-inspired bronchoscopic image segmentation for lung cancer diagnosis

**DOI:** 10.3389/fonc.2026.1706202

**Published:** 2026-02-20

**Authors:** Qian Li, Xinbo Liu, Chao Ye, Sen Cui, Jinze Zhang, Xuanyu Meng, Jin Guo, Xianjun Min

**Affiliations:** 1 Department of Thoracic Surgery, Beijing Genertec Aerospace Hospital, Beijing, China; 2 Department of Thoracic Surgery the Fourth Hospital of Hebei Medical University, Shijiazhuang, China; 3 School of Pharmacy, Hebei Medical University, Shijiazhuang, China; 4 School of Computer Science, Hunan University of Technology and Business, Changsha, Hunan, China; 5 China General Technology Group, Strategy Planning & Consulting Department, Beijing, China; 6 School of Medical Technology, Beijing Institute of Technology, Beijing, China

**Keywords:** adapter-based deep learning, bronchoscopic imaging, lesion segmentation, lung cancer diagnosis, multitask learning

## Abstract

**Introduction:**

Lung cancer remains the leading cause of cancer-related mortality. Although bronchoscopy allows direct visualization and tissue sampling, detecting subtle lesions is still challenging owing to limited resolution, variable imaging conditions, and the complex structure of the airway. Most existing approaches treat lesion segmentation and cancer diagnosis as separate tasks, which can reduce diagnostic coherence and limit clinical applicability.

**Method:**

We propose a novel Multi-Adapter-based Segment Any Bronchoscope Model (MASA), an end-to-end framework with an encoder that fuses spatial, frequency, and positional information and a dual decoder that performs simultaneous lesion segmentation and lung cancer diagnosis. MASA was trained/evaluated on the public BM-BronchoLC dataset.

**Results:**

On BM-BronchoLC, MASA improved lesion segmentation over the strongest baseline (ESFPNet), raising mean Dice coefficient (mDice) by +3.01% and mean Intersection-over-Union (mIoU) by +1.24%. For diagnosis, MASA increased Macro-F1 by +8.1 points and area under the precision–recall curve (AUPRC) by +14.1%.

**Conclusion:**

MASA provides a unified and interpretable pipeline for automated bronchoscopic image analysis, generating pixel-level lesion maps alongside case-level diagnostic predictions. The framework shows strong promise for improving early lung cancer detection and enhancing the efficiency of bronchoscopic workflows in clinical practice.

## 1 Introduction

Lung cancer remains the leading cause of cancer-related mortality worldwide (1–3). Early diagnosis is crucial, as 5-year survival exceeds 90% for Stage I disease but drops below 10% for Stage IV (4). Bronchoscopy is a vital tool for the detection and biopsy of suspicious lesions in the airways, playing a key role in diagnosing lung cancer and other serious respiratory conditions (5). However, bronchoscopic diagnosis is still largely a manual, operator-dependent process that suffers from considerable inter-observer variability (6). Even experienced bronchoscopists may miss subtle mucosal lesions or interpret findings inconsistently, leading to diagnostic delays and the potential for missed early cancers. These limitations underscore the need for computer-aided diagnosis (CAD) systems to assist clinicians during bronchoscopy (7).

Deep learning has dramatically advanced medical image analysis in recent years (8), exhibiting remarkable potential in bronchoscopic imaging. For instance, ESFPNet achieves real-time lesion segmentation in autofluorescence video, attaining a mean Dice score of 0.756 (9), while other studies using multi-scale attention report an expert-level accuracy of approximately 97% in malignancy classification (5). Despite these breakthroughs, two key challenges remain. First, reliable detection of fine-grained lesions is hampered by limited spatial resolution, motion blur, airway complexity, and variable mucosal appearance. Vision transformer–convolutional neural network (CNN) hybrids such as TransFuse (10) and DS-TransUNet (11) demonstrate enhanced multi-scale encoding but have not been extensively validated on bronchoscopic data. Second, most existing methods address segmentation and classification in isolation: segmentation networks like PraNet can delineate lesions precisely (12), but they do not provide malignancy prediction, while classification-only models lack accurate spatial localization (5). This separation complicates clinical integration, as spatial and diagnostic outputs must be manually reconciled.

These limitations motivate our research. Leveraging the rich lesion and malignancy annotations in the BM-BronchoLC dataset (13), we developed the Multi-Adapter-based Segment Any Bronchoscope Model (MASA), a unified framework for simultaneous lesion segmentation and lung cancer classification. MASA incorporates a context-aware encoder that integrates the Hierarchical Overlap-aware Patch Encoder (HOPE), the Patch and Ultra-frequency Learned Semantic Enhancer (PULSE), and the Positional Embedding Aggregated Knowledge (PEAK) modules, enabling the joint fusion of spatial, frequency, and positional information. In parallel, a dual-task Lesion-Unified Classification and Image Decoding (LUCID) decoder enables seamless multi-task prediction. By consolidating localization and diagnosis into a single pipeline, MASA provides consistent, interpretable, and clinically actionable outputs, addressing a critical need for computer-aided bronchoscopy.

The main contributions of our work are as follows:

1. Firstly, we propose the MASA, a fully integrated system for bronchoscopy that performs both lesion segmentation and lung cancer diagnosis within a single pipeline.
2. Secondly, we design a novel multi-adapter feature encoder that jointly integrates multi-scale spatial information, frequency-domain cues, and positional context to construct enriched, hierarchically informed feature representations. By adaptively capturing both fine-grained details and global structural patterns, this encoder significantly enhances the model’s sensitivity to subtle lesions in bronchoscopic images.
3. Thirdly, we devised a LUCID module that simultaneously produced segmentation masks and classification outputs. Through cooperative optimization on shared encoder representations while keeping task-specific decoders independent, the dual-task design yielded accurate and reliable diagnostic results and streamlined the clinical workflow.
4. Finally, MASA was validated on the public BM-BronchoLC dataset. Results indicated that MASA outperformed the compared baselines U-Net++ and ESFPNet.

## 2 Related work

### 2.1 Bronchoscopic image analysis with deep learning

Recent years have seen growing research into artificial intelligence (AI)-assisted bronchoscopy (5, 14, 15). Early deep learning models focused on classifying bronchoscopic findings, attaining impressive accuracies. Sun et al. (5), for example, reported over 97% accuracy in distinguishing malignant from benign bronchoscopic images using a multiscale attention CNN, approaching the performance of expert pulmonologists. Deng et al. (15) developed a deep recognition model to differentiate lung cancer subtypes in bronchoscopic views, while Yan et al. (16) proposed a knowledge-distillation network (PKDN) that infused prior medical knowledge to enhance bronchoscopic image classification. Beyond supervised learning, unsupervised anomaly detection has also been explored. For instance, Liu et al. (17) introduced a memory-augmented autoencoder that learns normal bronchoscopy appearances and flags deviations (tumors) without manual labels, achieving high sensitivity for intratracheal lesion detection. In the gastrointestinal (GI) endoscopy domain, AI systems have already demonstrated clinical value in detecting lesions: deep learning models can localize colorectal polyps and early GI cancers in real time with expert-level sensitivity (18, 19), and such systems are beginning to assist clinicians in practice (20). These successes in related fields underline the potential for AI to improve bronchoscopic diagnostics, provided the unique challenges of airway imagery are addressed.

However, most existing approaches remain constrained by suboptimal performance on subtle or small lesions, limited robustness to image artifacts (e.g., motion blur, specular highlights, and uneven illumination), and a lack of integrated frameworks capable of jointly performing lesion localization and pathological diagnosis. These shortcomings underscore the need for more advanced, unified deep learning models specifically designed to address the unique challenges of bronchoscopic imaging.

### 2.2 Segmentation techniques in endoscopic imaging

Lesion segmentation is a critical component of endoscopic image analysis. CNN architectures like U-Net (21) have become ubiquitous for medical image segmentation, including in endoscopy. U-Net++ (22) and other variants introduce redesigned skip connections and nested decoders to better capture multi-scale features, which has proven effective for segmenting lesions in complex scenes. For example, Wang et al. (23) combined multi-scale context and attention to improve GI lesion segmentation performance. Advanced attention mechanisms—such as the reverse attention in PraNet for polyp segmentation (12)—further refine the delineation of subtle lesions against surrounding mucosa. More recently, transformer-based architectures have been adopted to capture global context in segmentation. Chen et al. proposed TransUNet, which integrates vision transformers as encoders to enhance long-range feature modeling (24). In bronchoscopy, Chang et al. (9) introduced ESFPNet, an efficient transformer-based network that achieves real-time segmentation of airway lesions in autofluorescence bronchoscope videos. Sun et al. (25) developed a deep learning system using a Multiscale Attention Residual Network (MARN) for analyzing bronchoscopic images. MARN incorporates a Multiscale Convolutional Block Attention Module (MCBAM) to better capture lesion-relevant spatial and channel features, and integrates Grad-CAM for improved interpretability. Tang et al. (26) proposed TransMT-Net, a multi-task network combining transformers for global context and CNNs for local details to jointly classify and segment GI endoscopic images. Integrated active learning mitigates limited labeled data. Evaluated on a composite dataset from CVC-ClinicDB, Macau Kiang Wu Hospital, and Zhongshan Hospital, it effectively leverages sparse annotations for accurate lesion identification and delineation. Despite these developments, conventional segmentation methods alone output only a binary mask; without an integrated diagnosis, a segmented lesion still requires a separate classification step by either an algorithm or a physician.

### 2.3 Multi-task learning for segmentation and classification

To provide comprehensive assistance, there is a growing interest in multi-task learning frameworks that jointly perform lesion segmentation and disease classification. The motivation is that segmentation can provide precise localization to inform classification, while classification imparts semantic context that can prioritize relevant regions for segmentation. Tang et al. (26) demonstrated this synergy in GI endoscopy by proposing a transformer-based multi-task network (TransMT-Net) that achieved 96.9 classification accuracy and 77.8% Dice for segmentation, outperforming separate single-task models. Generally, multi-task deep learning can improve data efficiency and generalization by sharing representations between tasks (27). In bronchoscopy, the recently released BM-BronchoLC dataset has enabled initial explorations of joint learning: Vu et al. (13) reported baseline models that perform simultaneous anatomical landmark segmentation and lesion classification. These works indicate that an integrated approach can yield more robust performance, supporting the design of end-to-end systems that align with real clinical workflows. MASA builds upon this idea, uniquely coupling segmentation and malignancy prediction for lung cancer assessment within one model.

Building on this foundation, we propose MASA—a unified deep learning framework that uniquely couples pixel-level lesion segmentation with image-level lung cancer diagnosis within a single architecture. In this work, we specifically aim to address key limitations of existing methods by enhancing sensitivity to subtle or small lesions, improving robustness against common bronchoscopic artifacts (e.g., motion blur, specular highlights, and uneven illumination), and delivering clinically interpretable outputs that support reliable, real-time decision-making during bronchoscopy.

## 3 Proposed approach

### 3.1 Overview

**Figure 1:**
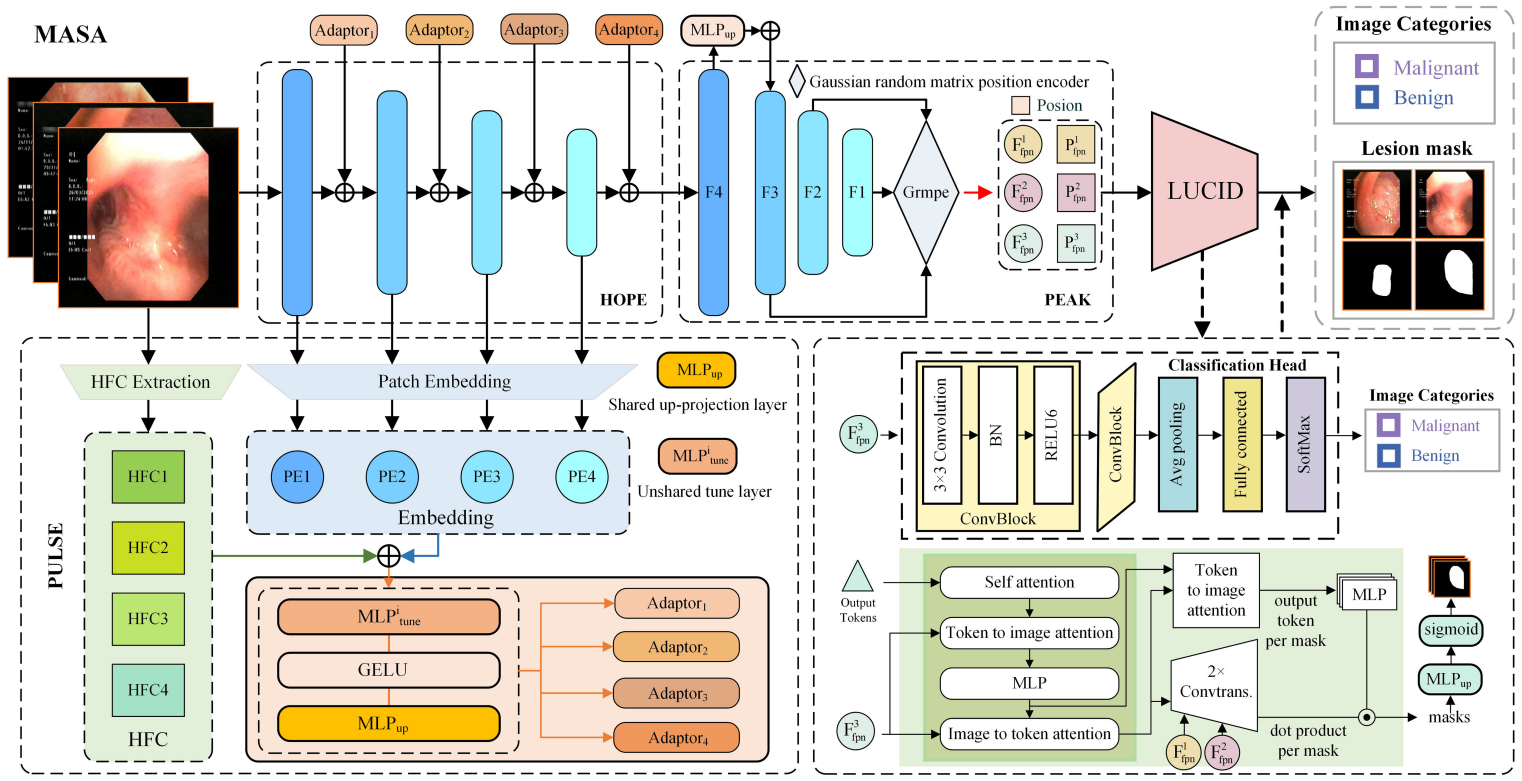
Schematic of the proposed MASA framework, consisting of four modules: HOPE for hierarchical feature extraction, PULSE for residual prompt generation, PEAK for multi-scale positional fusion, and LUCID for joint segmentation and classification from bronchoscopic images.

In this study, we propose MASA for lung cancer diagnosis, a unified end-to-end framework that consists of a Multi-Adapter Hierarchical Spatial-Frequency Encoder and a LUCID decoder. As illustrated in **Figure 1**, the encoder integrates three specialized modules: the HOPE module for extracting multi-scale spatial features, the PULSE module for frequency-domain enhancement, and the PEAK module for positional context aggregation. The decoder, implemented as the LUCID module, simultaneously generates precise lesion segmentation masks and lung cancer diagnostic predictions. This comprehensive design addresses the core challenges of bronchoscopic imaging and offers substantial clinical value by improving diagnostic accuracy and supporting clinical decision-making.

### 3.2 Multi-adapter hierarchical spatial-frequency encoder

#### 3.2.1 Hierarchical overlap-aware patch encoder

The MASA pipeline begins with the HOPE module, which conducts low-level feature extraction through a hierarchical transformer backbone. Specifically, we instantiated Hiera, a recent hierarchical vision transformer (28), to generate a pyramid of feature maps at multiple resolutions. The overlap patch embedding layers in Hiera divide the bronchoscopic image into overlapping patches, preserving continuity of local tissue structures at patch boundaries. By leveraging self-attention within and across hierarchical scales, the backbone yields rich multi-scale representations that encompass both fine-grained mucosal details and broader bronchial context.

Formally, for an input bronchoscopic image $I\in {\mathbb{R}}^{H_{0}\times W_{0}\times 3}$, the HOPE module produces feature maps $F^{s}$ at S hierarchical levels, where s=1, 2, 3, 4:

(1) $$F^{s}={\text{Hiera}}_{s}\left(I\right)$$

where each $F^{s}$ has spatial dimensions $H_{s}\times W_{s}$ and $C_{s}$ channels. The resolution decreases and the channel capacity increases with network depth, enabling early layer ( $F^{1},F^{2}$) to capture fine textures and lesion boundaries, while deeper layers ( $F^{3},F^{4}$) encode more abstract and global anatomical information.

#### 3.2.2 Patch and ultra-frequency learned semantic enhancer

To further enhance lesion sensitivity in bronchoscopic imaging under challenging conditions such as low illumination, motion blur, and specular highlights from moist mucosa, MASA introduces the PULSE module. This component generates frequency-aware residual prompts that adaptively modulate HOPE features, enabling the network to focus on lesion-relevant edges and texture variations by incorporating high-frequency visual information.

We derive a high-frequency enhancement signal from the input image via frequency filtering. Let ℱ and ℱ^−1^ denote the Fourier and inverse Fourier transforms, respectively. We first compute the image’s frequency spectrum S=ℱ(I), with zero-frequency component shifted to the center. To isolate the high-frequency components of the image spectrum, a binary mask *B_m_*(*u,v*) is applied to suppress the central low-frequency region, which corresponds to a small proportion *m* of the total spectral power. Specifically, the mask is defined as:

(2) $$B_{m}\left(u,v\right)=\{\begin{array}{ll} 0, & \text{if }\frac{4\left|\left(u-\frac{H}{2}\right)\left(v-\frac{W}{2}\right)\right|}{HW}\leq m\\ 1, & \text{otherwise} \end{array}$$

This formulation eliminates the low-frequency components from the frequency domain representation S=ℱ(I) by setting the corresponding coefficients to zero, thereby emphasizing edge-like and textural details in subsequent processing. The high-frequency component image

$I_{HFC}$ is then obtained by filtering and transforming back:

(3) $$I_{HFC}={ℱ}^{-1}\left(B_{m}\left(u,v\right),S\right)$$

The result $I_{HFC}$ preserves only the rapid intensity variations from *I*, which are often indicative of lesion boundaries and texture.

Next, PULSE uses learnable adapter networks to fuse this frequency information with the semantic features from HOPE. For each scale s in the hierarchy, we construct a residual prompt $R^{s}$ as follows. First, the corresponding HOPE feature $F^{s}$ is passed through a small tuning layer to reduce its dimension (controlling the number of parameters). In our design, we use a linear projection that compresses $F^{s}$ by a factor r to produce a patch embedding cue $E_{PE}^{s}={\text{L}}_{\text{PE}}^{\text{s}}\left(F^{s}\right)$. This focuses the high-level semantic content of $F^{s}$ into a compact embedding. In parallel, we extract a similarly shaped frequency embedding $E_{HFC}^{s}$ from the high-frequency image $I_{HFC}$. This is achieved by feeding $I_{HFC}$ through the same overlapping patch embed and transformer layers up to level s, yielding an embedding that aligns spatially with $F^{s}$. The two embeddings $E_{PE}^{s}$ and $E_{\text{HFC}}^{s}$ are then combined by a learnable adapter. We simply add them and apply a nonlinearity, then project back to the original channel dimension $C_{s}$:

(4) $$R^{s}=\rho \left(\text{GELU}\left(\phi \left(E_{PE}^{s}+E_{HFC}^{s}\right)\right)\right)$$

where GELU(·) denotes the activation, and both *ρ* and *ϕ* are up-projection operators that map the fused embedding back to *C_s_* channels. The adapter parameters are trained to produce an effective prompt for each transformer stage.

Finally, the residual prompt injection is performed: at each hierarchy level *s*, the prompt $R^{s}$ is added to the original feature map *F^s^* before it is passed to the next network stage:

(5) $$F^{s}=F^{s}+R^{s}$$

In summary, $R^{s}$ functions as a learned residual that guides the model’s attention toward lesion-specific frequencies and patterns at each hierarchy level. This design allows PULSE to enhance the encoding of subtle edges and textural details essential for accurate bronchoscopic lesion detection.

#### 3.2.3 Positional embedding aggregated knowledge

Following feature extraction by HOPE and frequency-enhanced prompting from PULSE, the PEAK module aggregates semantic information across scales and injects spatial awareness to support accurate lesion delineation in bronchoscopic imagery. Lesions in such images often exhibit subtle boundaries and variable morphology, requiring both localized texture analysis and global anatomical context. PEAK addresses these needs through a twofold strategy: top-down semantic feature fusion and learnable positional encoding.

To enrich spatial detail with deep semantic context, PEAK performs a selective top-down fusion. Classic Feature Pyramid Networks (FPNs) build semantic feature maps at every scale via a full top-down pathway with lateral connections (29). In contrast, PEAK transfers semantic information only from the deepest feature layer to its immediately preceding layer. This focused approach retains multi-scale semantics while reducing complexity.

Let $F^{s}$ denote the output feature at level *s*, with increasing *s* corresponding to lower spatial resolution and higher semantic abstraction. We define the fused feature $F_{\text{FPN}}^{s}$ as:

(6) $$F_{FPN}^{s}=\{\begin{array}{ll} F^{s}+\text{U}\left(F^{s+1}\right), & s=3\\ F^{s}, & s<3 \end{array}$$

Here, U(·) denotes nearest-neighbor interpolation to match spatial resolution. This formulation allows the highest-level feature $F^{4}$, which contains the most abstract lesion semantics, to influence level $F^{3}$ while leaving the finer-scale levels $F^{1}$ and $F^{2}$ structurally intact.

After fusion, the deepest layer is discarded to avoid redundancy, and the final fused outputs are defined as $\left\{F_{FPN}^{1},F_{FPN}^{2},F_{FPN}^{3}\right\}$. This design ensures that high-resolution detail is preserved while incorporating selective semantic guidance, enabling the model to better differentiate subtle lesion textures from artifacts or airway bifurcations.

To further augment spatial coherence, PEAK assigns positional encodings to each fused feature map. These encodings help the network interpret anatomical location and orientation, which are essential for distinguishing between airway zones in bronchoscopic views.

Given a pixel location (u,v), normalized to the range [−1, 1], we compute its positional embedding using the following formulation:

(7) $$\text{PE}\left(u,v\right)=\left[\text{sin }\left(2\pi G\cdot {\left[u,v\right]}^{T}\right),\text{cos }\left(2\pi G\cdot {\left[u,v\right]}^{T}\right)\right]$$

Here, $G\in {\mathbb{R}}^{d\times 2}$ is a learnable Gaussian matrix, randomly initialized and optimized during training. Each fused feature $F_{FPN}^{i}$ is paired with its positional embedding, forming three aligned pairs: $\left(F_{FPN}^{1},P_{FPN}^{1}\right),$ $\left(F_{FPN}^{2},P_{FPN}^{2}\right)$, and $\left(F_{FPN}^{3},P_{FPN}^{3}\right)$. These outputs carry both texture-rich and position-aware representations across multiple scales, ready to be decoded into segmentation masks and image-level classifications.

### 3.3 Lesion-unified classification and image decoding

The LUCID module converts the multi-scale features generated by PEAK into two outputs: a high-resolution binary segmentation mask and an image-level lung cancer diagnosis. Designed as a dual-headed decoder, the LUCID module enables efficient multi-task learning by sharing feature representations while maintaining task-specific processing.

Image-level classification head. For lung cancer diagnosis, LUCID extracts the lowest-resolution fused feature from PEAK, which contains the most informative semantic abstraction. This feature is first upsampled to enhance spatial detail and then passed through a lightweight classification network comprising sequential convolutional layers, batch normalization, activation, global average pooling, and a fully connected layer. The classification process is defined as:

(8) $$y_{\text{cls}}=\text{Classifier}\left(\text{U}\left(F_{\text{cls}}\right)\right)$$

where $F_{\text{cls}}$ denotes the lowest-resolution fused feature from PEAK, U(·) represents the upsampling operation, and Classifier(·) encapsulates the classification network. During training, classification gradients are detached from the encoder to avoid interference with segmentation learning.

Lesion segmentation decoder. The segmentation branch of LUCID builds on a modified prompt-based decoding approach; it converts feature representations into pixel-level binary lesion masks via a transformer-guided decoding process. This process involves token-conditioned spatial interaction, multi-scale upsampling, and mask generation using dynamic weight prediction.

We initialize a learnable dense prompt embedding $E_{\text{dense}}$ and inject it into the deepest semantic feature from PEAK, denoted as $F_{FPN}^{3}$. The two are combined to produce the input spatial feature for the transformer:

(9) $$S=E_{\text{dense}}+F_{FPN}^{3}$$

We then define an initial set of learnable mask tokens $T_{0}\in {\mathbb{R}}^{M\times d}$, where M=1 by default but can be generalized to any number of output masks. Along with these, we introduce continuous positional encoding $E=P_{FPN}^{3}$. The triplet $\left(S,T_{0},PE\right)$ is passed into a Two-Way Transformer to enable bidirectional conditioning:

(10) $$S^{\prime },T_{M}=\text{TwoWayTrans}\left(S,T_{0},PE\right)$$

where $S^{\prime }$ is the updated spatial feature and $T_{M}$*is* the refined token set.

To progressively recover the full spatial resolution and refine features with earlier-scale information, we apply two stages of upsampling and feature fusion. The first upsampling output is computed as:

(11) $$U_{1}=\text{GELU}\;(\text{LN}\;(\text{ConvTrans}\left(S^{\prime }\right)+F_{FPN}^{2}))$$

Here, ConvTrans is a transposed convolution that halves the channel dimension and doubles the spatial size; *LN* denotes Layer Norm. The second upsampling and fusion stage yields:

(12) $$U_{2}=\text{GELU}\;(\text{ConvTrans}\left(U_{1}\right)+F_{FPN}^{1}),\;U_{2}\in {\mathbb{R}}^{H\times W\times d}$$

For each refined mask token $T_{i}\in T_{M},$ we apply a lightweight MLP to produce a query vector $W_{i}\in {\mathbb{R}}^{d}$. This vector interacts with the spatial embedding $U_{2}\in {\mathbb{R}}^{H\times W\times d}$, which is first flattened to ${\hat{U}}_{2}\in {\mathbb{R}}^{d\times \left(H\cdot W\right)}$. The dot product at each spatial location yields the mask logits:

(13) $$M_{i}=w_{i}^{⊤}\cdot {\hat{U}}_{2}$$

The resulting logit map $M_{i}\in {\mathbb{R}}^{H\times W}$ is then bilinearly upsampled to match the original input resolution $\left(H_{0},W_{0}\right)$:

(14) $$M_{i}^{up}=\text{U}\left(M_{i},\left(H_{0},W_{0}\right)\right)$$

A pixel-wise sigmoid function is applied to obtain the final probability mask:

(15) $$P_{\text{mask}}\left(x,y\right)=\frac{1}{1+\text{exp}\left(-M_{i}^{up}\left(x,y\right)\right)}$$

Finally, the probability mask is binarized using a fixed threshold to generate the final segmentation output M(x,y). This decoder architecture facilitates accurate lesion delineation by fusing coarse semantic and fine spatial features under internal prompt guidance, producing detailed segmentation masks that align with clinical bronchoscopic imagery.

### 3.4 Dual-task cooperative optimization

To jointly optimize segmentation and classification performance, we adopted a multi-objective loss composed of pixel-level and image-level terms.

To penalize pixel-level classification errors, the Binary Cross-Entropy (BCE) loss is defined as:

(16) $$L_{\text{BCE}}=-\frac{1}{N}\sum_{p=1}^{N}\left[y_{p}\cdot \text{log }\sigma \left(z_{p}\right)+\left(1-y_{p}\right)\cdot \text{log}\left(1-\sigma \left(z_{p}\right)\right)\right]$$

where N is the number of pixels, $y_{p}\in \left\{0,\;1\right\}$ denotes ground truth, and $z_{p}$ is the predicted logit at pixel p.

To improve lesion boundary localization, we incorporate the soft Intersection-over-Union (IoU) loss:

(17) $$L_{\text{IoU}}=1-\frac{\sum_{i}p_{i}y_{i}}{\sum_{i}(p_{i}+y_{i}-p_{i}y_{i})}$$

with $p_{i}=\sigma \left(z_{i}\right)$ as the predicted probability and $y_{i}$ as the binary ground truth.

The overall segmentation loss combines BCE and IoU with empirically chosen weights:

(18) $$L_{\text{seg}}=\lambda_{\text{BCE}}\cdot L_{\text{BCE}}+\lambda_{\text{IoU}}\cdot L_{\text{IoU}}$$

The classification supervision employs a standard cross-entropy loss:

(19) $$L_{\text{cls}}=-\left[y_{\text{cls}}\text{log }\hat{p}+\left(1-y_{\text{cls}}\right)\;\text{log}\left(1-\hat{p}\right)\right],\;\hat{p}=\sigma \left(q\right)$$

where q∈ℝ is the output logit from the classification branch, and $y_{\text{cls}}\in \left\{0,\;1\right\}$ is the ground-truth malignancy label.

Finally, the total multi-task loss is expressed as:

(20) $$L_{\text{total}}=\lambda_{\text{seg}}\cdot L_{\text{seg}}+\lambda_{\text{cls}}\cdot L_{\text{cls}}$$

where $\lambda_{\text{seg}}$, $\lambda_{\text{cls}}$, $\lambda_{\text{BCE}}$, and $\lambda_{\text{IoU}}$ are empirically chosen weights balancing segmentation and classification objectives.

This cooperative loss structure ensures that the network learns both local lesion boundaries and global diagnostic signals in a mutually reinforcing manner. Here should be added a sentence. The overall optimization is summarized in **Algorithm 1**.

Algorithm 1

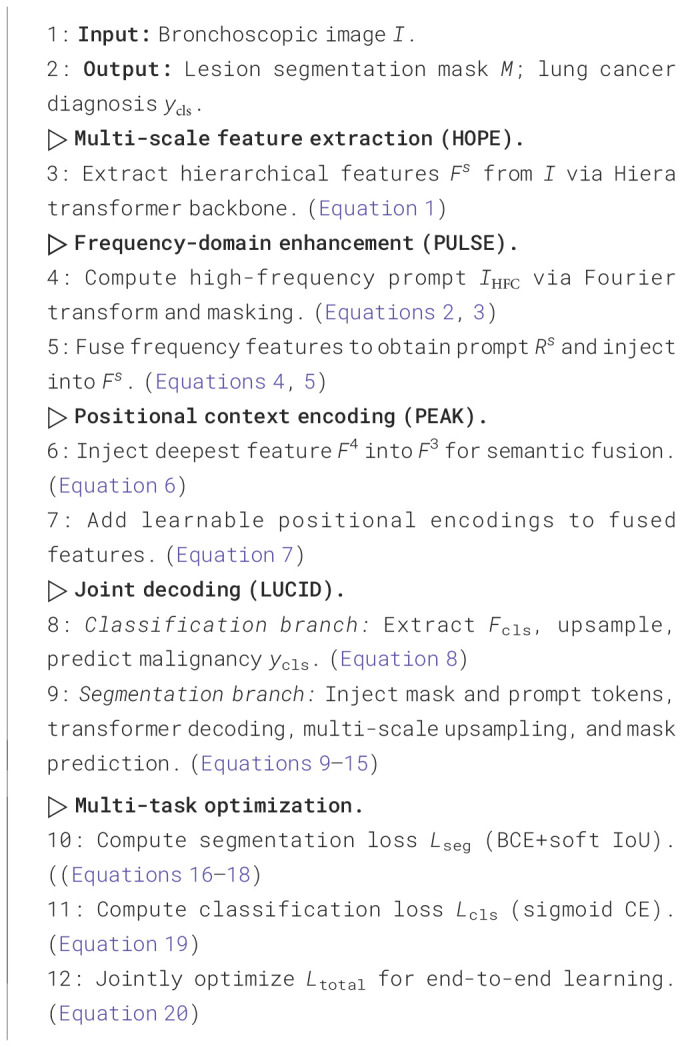

## 4 Experiments

### 4.1 Dataset

The performance of the proposed MASA framework was comprehensively evaluated on the public BM-BronchoLC dataset (13), which provides high-resolution bronchoscopic images annotated with pixel-level lesion masks and verified malignancy labels. Two clinically significant tasks were investigated: (1) precise segmentation of lesion regions and (2) binary classification of each lesion as malignant (lung cancer) or benign (non-lung cancer). Low-quality or ambiguously annotated images were excluded to ensure the reliability of the experimental data. The BM-BronchoLC clinical cohort consists of 208 patients with a total of 2,921 images, collected from Bach Mai Hospital (Vietnam) between 2020 and 2023. All images were acquired from flexible bronchoscopy examinations using standard white-light imaging. Bronchoscopy videos were recorded and representative frames were extracted at 1 frame per second, after which experienced bronchoscopists selected at least 10 high-quality images per patient for annotation. The final curated dataset includes 2,132 images containing anatomical landmarks and 789 images containing lung-cancer-related lesions.

For all experiments, the dataset was partitioned into training, validation, and test subsets to enable rigorous and unbiased evaluation by 10-fold cross-validation. Specifically, approximately 80% of the images were allocated for training, 10% for validation, and 10% for testing. **Table 1** summarizes the distribution of malignant and benign lesions across these subsets.

**Table 1:** Distribution of lesion samples in the BM-BronchoLC dataset.

Label	Train set	Val set	Test set	Total
Lung cancer	505	59	63	627
Non-lung cancer	85	7	10	102
Total	590	66	73	729

### 4.2 Evaluation metrics

For a comprehensive evaluation, we adopted several widely recognized quantitative metrics for both classification and segmentation tasks. All metrics are computed with the malignant (lung cancer) category as the positive class.

#### 4.2.1 Classification metrics

The classification performance is assessed using accuracy (Equation 21), precision (Equation 22), recall (Equation 23), and F1-score (Equation 24). Their mathematical definitions are as follows:

(21) $$\text{Accuracy}=\frac{TP+TN}{TP+TN+FP+FN}$$

(22) $$\text{Precision}=\frac{TP}{TP+FP}$$

(23) $$\text{Recall}=\frac{TP}{TP+FN}$$

(24) $$\text{F}1-\text{score}=\frac{2TP}{2TP+FP+FN}$$

where TP, FP, TN, and FN represent the number of true positives, false positives, true negatives, and false negatives, respectively.

The area under the precision–recall curve (AUPRC) is then computed as the integral of precision with respect to recall (Equation 25):

(25) $$\text{AUPRC}=\int_{0}^{1}\text{Precision}\left(r\right)\;d\mathrm{r.}$$

#### 4.2.2 Segmentation metrics

For lesion segmentation, six quantitative metrics were adopted: overall Accuracy (oAcc) (Equation 26), mean Intersection-over-Union (mIoU) (Equation 28), mean Accuracy (mAcc) (Equation 27), mean Dice coefficient (mDice) (Equation 29), mean Precision (mPrec) (Equation 30), and mean Recall (mRecall) (Equation 31). These metrics are defined as follows:

(26) $$\text{oAcc}=\frac{\sum_{i=1}^{C}p_{ii}}{\sum_{i=1}^{C}\sum_{j=1}^{C}p_{ij}}$$

(27) $$\text{mAcc}=\frac{1}{C}\sum_{i=1}^{C}\frac{p_{ii}}{\sum_{j=1}^{C}p_{ij}}$$

(28) $$\text{mIoU}=\frac{1}{C}\sum_{i=1}^{C}\frac{p_{ii}}{\sum_{j=1}^{C}p_{ij}+\sum_{j=1}^{C}p_{ji}-p_{ii}}$$

(29) $$\text{mDice}=\frac{1}{C}\sum_{i=1}^{C}\frac{2p_{ii}}{2p_{ii}+\sum_{j=1,j\neq i}^{C}(p_{ij}+p_{ji})}$$

(30) $$\text{mPrec}=\frac{1}{C}\sum_{i=1}^{C}\frac{p_{ii}}{\sum_{j=1}^{C}p_{ji}}$$

(31) $$\text{mRecall}=\frac{1}{C}\sum_{i=1}^{C}\frac{p_{ii}}{\sum_{j=1}^{C}p_{ij}}$$

Here, C represents the total number of classes, which is two in this study (lesion and background). $p_{ij}$ denotes the number of pixels of class i that are predicted as class j, $p_{ii}$ indicates the number of pixels correctly predicted as class i, $\sum_{j}p_{ij}$ is the total number of pixels belonging to class i in the ground truth, and $\sum_{j}p_{ji}$ is the total number of pixels predicted as class i. Collectively, these metrics evaluate pixel-level agreement, region-level overlap, and the balance between sensitivity and precision for lesion delineation.

### 4.3 Implementation details

The MASA framework was implemented in PyTorch and trained on two NVIDIA RTX 4090 GPUs using distributed data parallelism. Optimization was performed with the AdamW optimizer, an initial learning rate of 2 × 10^−4^, and a batch size of 1. Training was conducted for up to 100 epochs with early stopping based on validation loss, and a cosine-annealing learning-rate schedule was adopted. The frequency mask ratio *m* in Equation 2 was set to 0.25. Within the segmentation loss defined in Equation 18, the weights for the BCE and IoU terms were set to $\lambda_{\text{BCE}}=1.0$ and $\lambda_{\text{seg}}=1.0$, respectively. For multi-task optimization in Equation 20, the segmentation and classification branches were weighted as λ_IoU_ = 2.0 $\;\text{and}\;\lambda_{\text{cls}}=1.8$. The training data were augmented using the MedAugment framework (30), which applies spatial transformations including rotation, horizontal and vertical flipping, scaling, translation along the *x*- and *y*-axes, and shearing in both directions prior to model fitting.

### 4.4 Classification task

To evaluate the effectiveness of MASA in automated lung cancer diagnosis, we conducted a comprehensive comparison on the binary classification of bronchoscopic lesions. The task is defined as distinguishing between lung cancer (malignant) and non-lung cancer (benign) based on bronchoscopic image features. All experiments were performed on the BM-BronchoLC dataset, using the metrics described in Section 4.2.

#### 4.4.1 Comparison with other advanced models

The class-wise performance of all compared methods on the BM-BronchoLC test set is summarized in **Table 2**. Given the pronounced class imbalance, 627 malignant versus 102 benign lesions evaluating models solely by oAcc would be misleading. Instead, we report per-class recall (sensitivity for malignant and specificity for benign), precision, macro-averaged F1-score, and the AUPRC for the benign (minority) class, which are more informative under such imbalance.

**Table 2:** Class-wise performance on the BM-BronchoLC test set. Macro-F1, AUPRC, and key clinical metrics are reported.

Model	Malignant		Benign		Macro-F1	AUPRC (benign)
	Recall	Precision	Recall	Precision	(%)	(%)
U-Net++	90.2 ± 0.23	91.7 ± 0.26	60.0 ± 0.22	54.5 ± 0.16	73.3 ± 0.26	58.2 ± 0.16
ESFPNet	93.4 ± 0.19	93.5 ± 0.21	70.0 ± 0.14	72.4 ± 0.21	82.1 ± 0.13	71.5 ± 0.1
MASA (ours)	98.3 ± 0.15	93.9 ± 0.09	80.0 ± 0.11	88.9 ± 0.08	90.2 ± 0.13	85.6 ± 0.07

U-Net++ achieves high recall (90.2%) and precision (91.7%) on malignant lesions but performs poorly on benign cases, with only 60.0% recall (i.e., specificity) and 54.5% precision. This implies that 40% of benign lesions are misclassified as malignant, potentially leading to a substantial number of unnecessary biopsies. ESFPNet improves upon this, attaining 70.0% benign recall and 72.4% benign precision, yet still mislabels 3 out of every 10 benign cases as malignant.

In contrast, our proposed method, MASA, demonstrates significant gains across all metrics. It achieves near-perfect sensitivity for malignancy (98.3% recall), missing only one malignant case in the test set—critical in clinical settings where false negatives carry severe consequences. More importantly, MASA substantially improves performance on the minority class: it correctly identifies 80.0% of benign lesions (specificity) with a high precision of 88.9%, meaning that among all lesions predicted as benign, nearly 89% are truly benign. This translates to only 2 false positives out of 10 benign cases, a notable reduction compared to baseline models. Consequently, MASA achieves the highest macro-F1 score (90.2%), reflecting balanced performance across both classes, and the highest AUPRC for the benign class (85.6%), confirming its superior ability to discriminate the minority class under precision–recall trade-offs.

These results indicate that MASA effectively mitigates the bias introduced by class imbalance while maintaining clinical safety: it minimizes missed cancers and simultaneously reduces unnecessary invasive procedures. The combination of high sensitivity and improved specificity makes MASA particularly suitable for real-world bronchoscopic lung cancer screening workflows.

These results underscore the advantages of MASA’s multi-adapter architecture and its cooperative optimization strategy. By leveraging shared multi-scale spatial, frequency, and positional representations while maintaining independent task-specific decoders, MASA generated more discriminative features and achieved superior diagnostic consistency compared with conventional single-task networks.

#### 4.4.2 Visual results of ROC analyses, confusion matrices, and t-SNE

**Figure 2:**
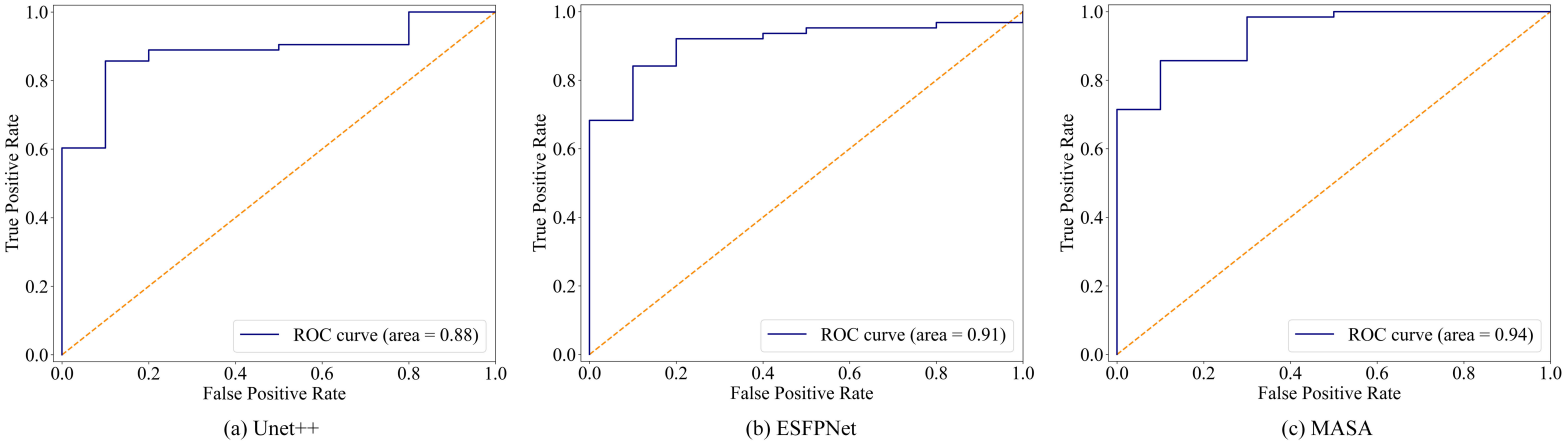
ROC curves of the proposed MASA and baseline classification models. **(a)** Unet++ with an area of 0.88, **(b)** ESFPNet with an area of 0.91, **(c)** MASA with an area of 0.94.

**Figure 3:**
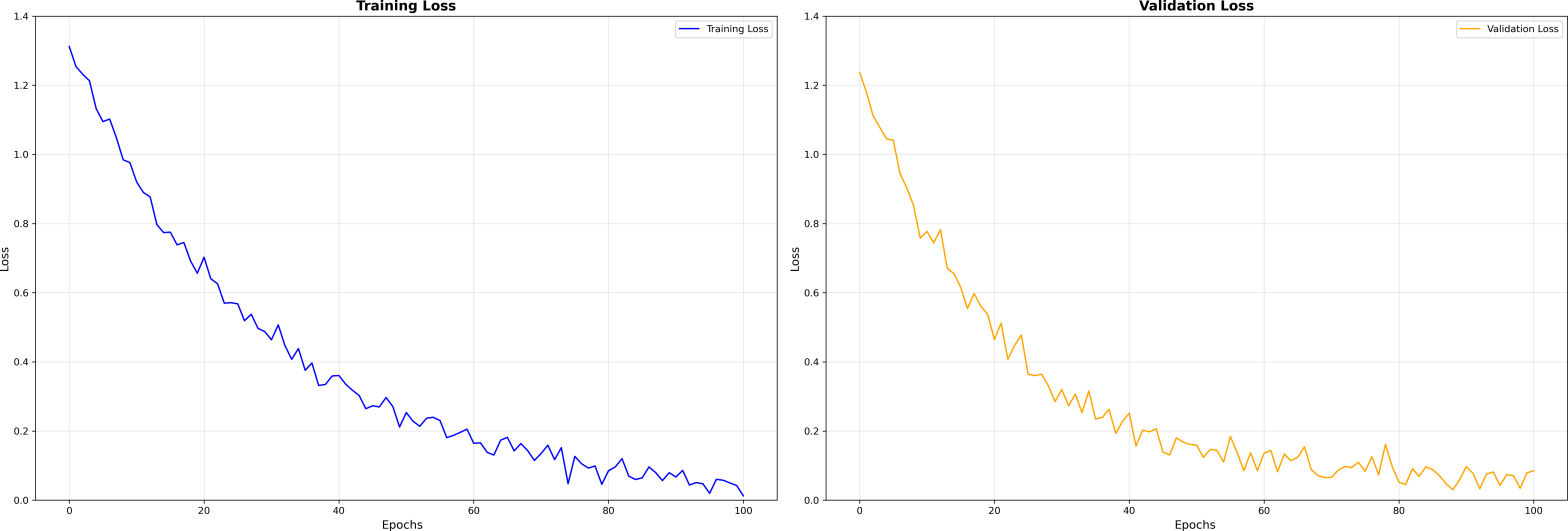
Training and validation loss.

Discriminative performance was characterized on the BM-BronchoLC test set with receiver operating characteristic analyses and confusion matrices. As shown in **Figure 2**, MASA achieved the largest area under the curve, AUC = 0.94, compared with ESFPNet at 0.91 and U-Net++ at 0.88. The upward shift of MASA’s curve across operating points indicated consistently stronger trade-offs between sensitivity and specificity for differentiating malignant from benign lesions. **Figure 3** shows training and validation loss over 100 epochs. Both curves decrease steadily, indicating effective learning. The validation loss remains slightly higher than training loss, typical for well-regularized models, but does not diverge or increase, suggesting no significant overfitting. The fluctuations in validation loss may stem from batch variability or a small validation set. Overall, the model converges well, with consistent improvement on both seen and unseen data, demonstrating stable training and generalization.

**Figure 4:**
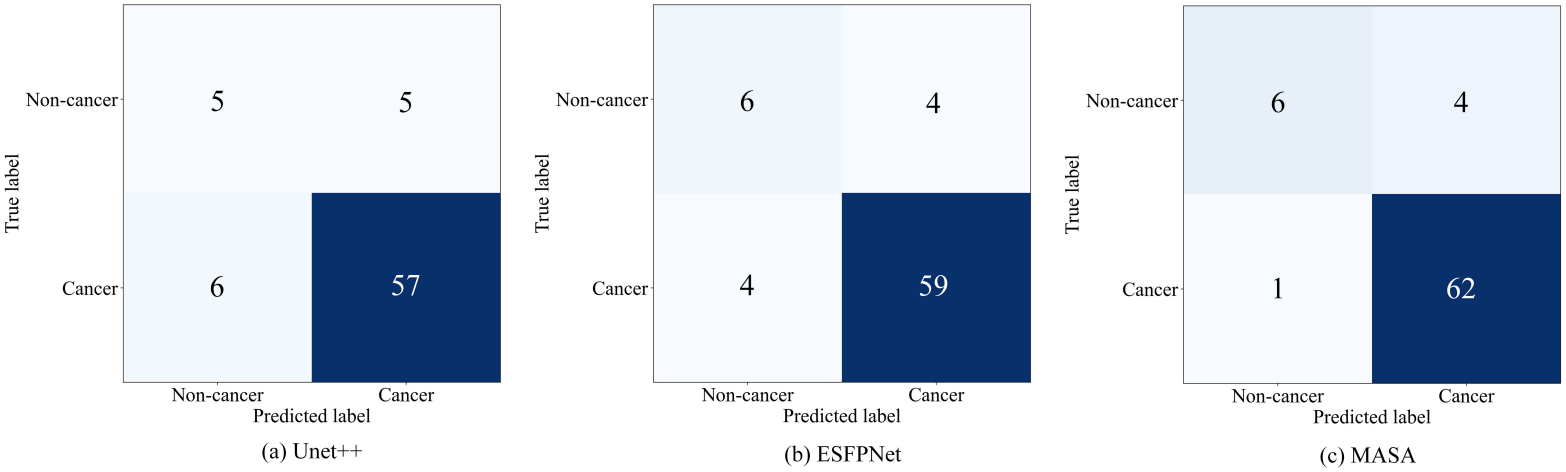
Confusion matrices of the proposed MASA and baseline classification models. **(a)** Unet++: True non-cancer 5, predicted as cancer 5, true cancer 6, predicted as cancer 57. **(b)** ESFPNet: True non-cancer 6, predicted as cancer 4, true cancer 4, predicted as cancer 59. **(c)** MASA: True non-cancer 6, predicted as cancer 4, true cancer 1, predicted as cancer 62.

**Figure 4** summarizes the confusion matrices at the operating point used for metric reporting. MASA markedly reduced missed cancers, with a false negative rate of 1 out of 63, or 1.6%, while maintaining a similar number of false positives to the baselines. This pattern was consistent with the higher recall and F1-score in **Table 2**.

**Figure 5:**
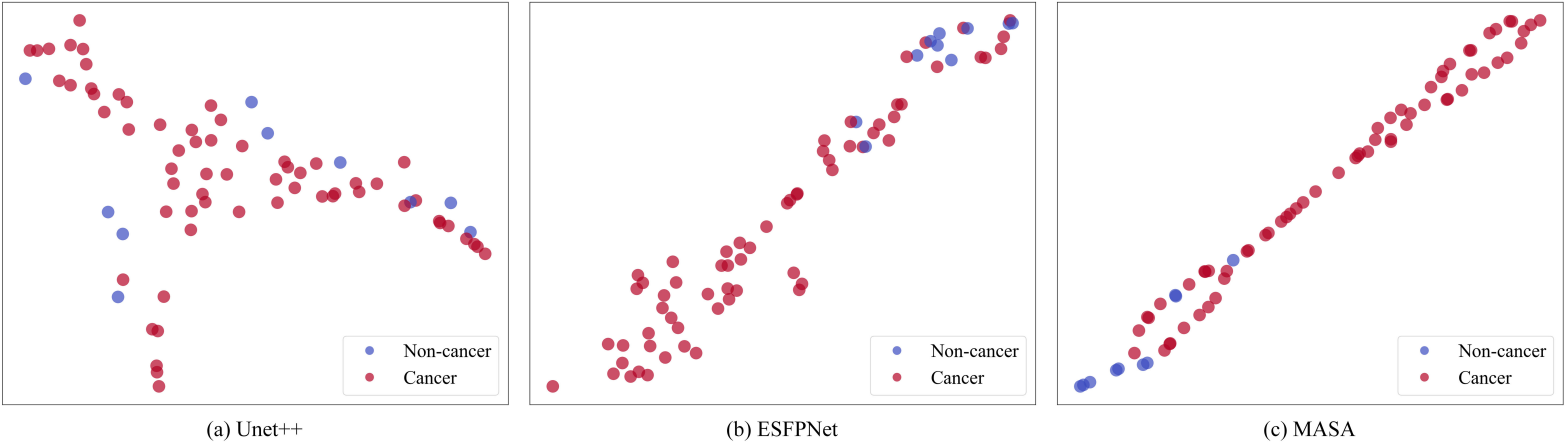
t-SNE visualization of feature distributions for MASA and baseline classification models. Panel **(a)** Unet++ shows mixed clustering of red and blue dots. Panel **(b)** ESPNet displays more distinct clustering, predominantly red dots. Panel **(c)** MASA shows a clear linear separation with red and blue dots. Blue represents non-cancer, red represents cancer.

To assess the separability of learned representations, **Figure 5** shows two-dimensional t-SNE projections of the feature embeddings. MASA formed tighter intra-class clusters and clearer inter-class boundaries, whereas U-Net++ and ESFPNet exhibited more overlap between classes. The improved separability corroborated the ROC and confusion matrix findings.

Collectively, these visual and quantitative results substantiate the effectiveness of the proposed multi-adapter architecture and cooperative optimization on shared multi-scale representations. By integrating spatial, frequency, and positional cues within a unified feature space while retaining independent task-specific decoders, MASA produced more discriminative features and demonstrated reliable image-level diagnosis. These properties indicate strong potential for deployment as decision support in bronchoscopic assessment of suspected lung cancer.

### 4.5 Ablation study

**Table 3** summarizes the impact of the three core submodules—HOPE, PEAK, and PULSE—on both segmentation and classification performance, evaluated using Macro-F1, AUPRC, mIoU, and mDice. All configurations retain the HOPE module, and the results illustrate the incremental benefits introduced by PEAK and PULSE.

**Table 3:** Performance comparison with/without different submodels (%).

HOPE	PEAK	PULSE	Macro-F1	AUPRC	mIoU	mDice
✔	×	×	80.36±0.43	73.36±0.37	33.11±0.33	49.01±0.14
✔	✔	×	83.63±0.22	76.39±0.26	36.37±0.31	50.30±0.24
✔	×	✔	86.29±0.16	80.30±0.21	40.66±0.14	52.22±0.26
✔	✔	✔	90.20±0.13	85.60±0.07	42.93 ± 0.11	56.74 ±0.09

(1) Baseline: HOPE only. With only the HOPE backbone enabled, the model obtains moderate performance (Macro-F1: 80.36%, AUPRC: 73.36%, mIoU: 33.11%, mDice: 49.01%). These results confirm that HOPE provides a solid foundational representation but lacks mechanisms for enhanced spatial refinement or temporal consistency. (2) Introducing PEAK on top of HOPE produces consistent improvements across all metrics, yielding gains of +3.27% in Macro-F1, +3.03% in AUPRC, + 3.26% in mIoU, and +1.29% in mDice. This indicates that the positional-aware fusion strategy in PEAK effectively strengthens spatial feature aggregation, enhancing lesion-focused attention and segmentation detail. (3) Replacing PEAK with PULSE (HOPE + PULSE) leads to even larger performance gains, achieving 86.29% Macro-F1 and 80.30% AUPRC, with substantial boosts in segmentation metrics (mIoU: 40.66%, mDice: 52.22%). The notable improvements—particularly the +7.55% increase in mIoU and +6.94% in AUPRC over the baseline—suggest that PULSE’s frequency-aware prompting plays a more dominant role in reinforcing lesion textures and boundaries. (4) The complete architecture yields the highest performance across all evaluation metrics, achieving 90.20% Macro-F1, 85.60% AUPRC, 42.93% mIoU, and 56.74% mDice. The continued gains over HOPE + PULSE (e.g., +4.52% in Macro-F1 and +2.27% in mIoU) highlight the complementary strengths of PEAK and PULSE. PEAK contributes fine-grained spatial refinement, while PULSE enhances high-frequency and semantic sensitivity; together, they form a balanced and synergistic encoder.

Overall, the interaction between PEAK and PULSE is essential for achieving state-of-the-art segmentation and classification performance in bronchoscopic imaging.

### 4.6 Segmentation task

To assess the efficacy of MASA for lesion delineation, we conducted a comprehensive evaluation on the BM-BronchoLC dataset. The objective was to accurately contour lesion regions in bronchoscopic images, which is central to diagnosis and interventional planning. All experiments adhered to the metrics defined in Section 4.2.

#### 4.6.1 Comparison with other advanced models

**Table 4** reports comprehensive lesion segmentation results on the BM-BronchoLC test set, comparing our proposed MASA framework against two strong baselines: U-Net++ (22) and ESF-PNet (9). All metrics are computed per lesion mask and averaged across the test set; “oAcc” denotes overall pixel accuracy, while “mIoU”, “mAcc”, “mDice”, “mPrec”, and “mRec” represent mean Intersection-over-Union, mean Accuracy, mean Dice coefficient, mean Precision, and mean Recall, respectively, averaged over the foreground (lesion) class.

**Table 4:** Comparison of lesion segmentation performance on the BM-BronchoLC test set.

Model	oAcc (%)	mIoU (%)	mAcc (%)	mDice (%)	mPrec (%)	mRec (%)
U-Net++	84.22 ± 0.26	36.57 ± 0.23	60.44 ± 0.24	48.19 ± 0.21	46.46 ± 0.16	63.28 ± 0.23
ESFPNet	85.56 ± 0.21	41.69 ± 0.20	68.39 ± 0.23	53.73 ± 0.19	51.05 ± 0.18	68.33 ± 0.16
MASA (ours)	86.99 ± 0.14	42.93 ± 0.11	71.13 ± 0.08	56.74 ± 0.09	54.77 ± 0.11	71.06 ± 0.13

U-Net++, while achieving a high overall pixel accuracy (84.22%), exhibits limited performance on the lesion region itself, with a low mIoU of 36.57% and a Dice of 48.19%. This discrepancy arises because oAcc is dominated by the abundant background pixels in bronchoscopic images, masking poor foreground localization. ESFPNet improves lesion-specific metrics notably—boosting mIoU to 41.69% and Dice to 53.73%—demonstrating better lesion delineation through its edge-enhanced features.

Our method, MASA, achieves consistent improvements across all lesion-centric metrics. It attains the highest mIoU (42.93%), mDice (56.74%), and mRec (71.06%), reflecting superior spatial alignment with ground-truth lesion boundaries and reduced under-segmentation. Notably, MASA also achieves the highest mPrec (54.77%), indicating tighter contour prediction with fewer false-positive regions compared to baselines. The gain in mAcc (71.13%) further confirms that MASA better balances correct foreground and background classification at the lesion level.

Although the absolute mIoU values may appear modest, this is characteristic of challenging medical segmentation tasks involving small, irregular, and low-contrast lesions in bronchoscopic video frames. Crucially, the 1.24% absolute improvement in mIoU over ESFPNet—and more substantial gains in Dice (3.01%) and Recall (2.73%)—translates to clinically meaningful enhancements in lesion coverage, which directly support downstream tasks such as targeted biopsy planning and longitudinal monitoring. The low standard deviations across repeated runs (≤0.14%) further attest to the stability of MASA’s segmentation output.

#### 4.6.2 Effect on frequency mask ratio

**Figure 6:**
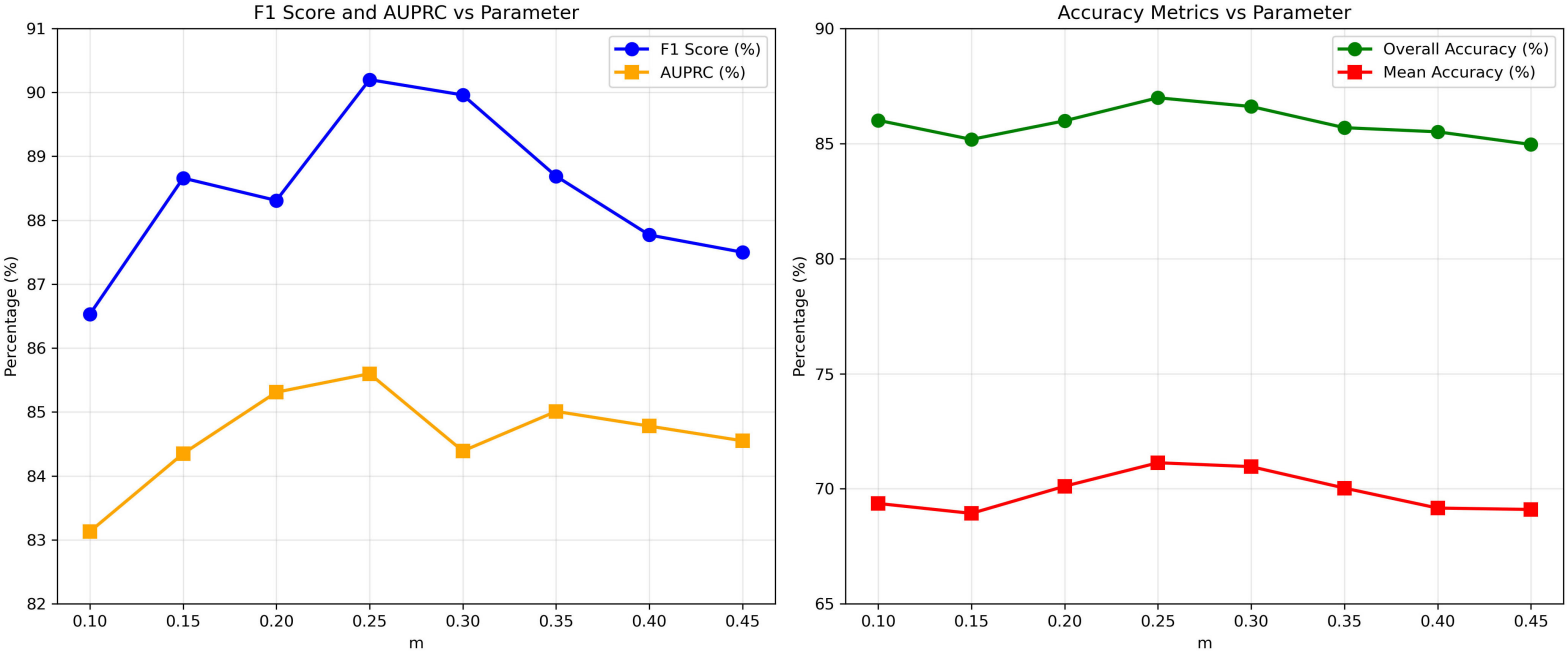
Analysis of frequency mask ratio (*m*).

We analyze the influence of the frequency mask ratio (*m*) on both classification and segmentation performance by varying *m* from 0.10 to 0.45. **Figure 6** reports the corresponding F1-score, AUPRC, oAcc, and mAcc.

As shown in **Figure 6** on the Macro-F1 and AUPRC metrics, performance first increases as *m* grows from 0.10 to 0.25 and then gradually declines for larger values. Specifically, the Macro-F1 score improves from 86.53% at *m* = 0.10 to a peak of 90.20% at *m* = 0.25, while AUPRC reaches its maximum value of 85.60% at the same setting. This trend indicates that moderate emphasis on high-frequency components effectively enhances discriminative cues relevant to classification. When *m >* 0.25, both Macro-F1 and AUPRC decrease, suggesting that overly aggressive masking may suppress informative mid-frequency structures or introduce noise into the representation.

A similar pattern is observed for segmentation-related metrics. The oAcc increases steadily from 86.01% at *m* = 0.10 to a maximum of 86.99% at *m* = 0.25, while the mAcc reaches its highest value (71.13%) at the same point. The subsequent decline in mAcc for larger *m* values indicates that excessive removal of low-frequency content can negatively impact class-wise segmentation consistency, particularly for regions that rely on global context rather than sharp boundaries.

Across both classification and segmentation tasks, the results consistently indicate that *m* = 0.25 provides the optimal balance between enhancing high-frequency structural information and preserving low- and mid-frequency semantic context. Smaller values of *m* fail to sufficiently highlight discriminative details, whereas larger values overemphasize high-frequency components, leading to performance degradation. These observations empirically justify our choice of *m* = 0.25 as the default configuration and demonstrate that the proposed frequency adapter is sensitive to controlled spectral emphasis rather than trivial high-pass filtering.

#### 4.6.3 Visual qualitative results

**Figure 7:**
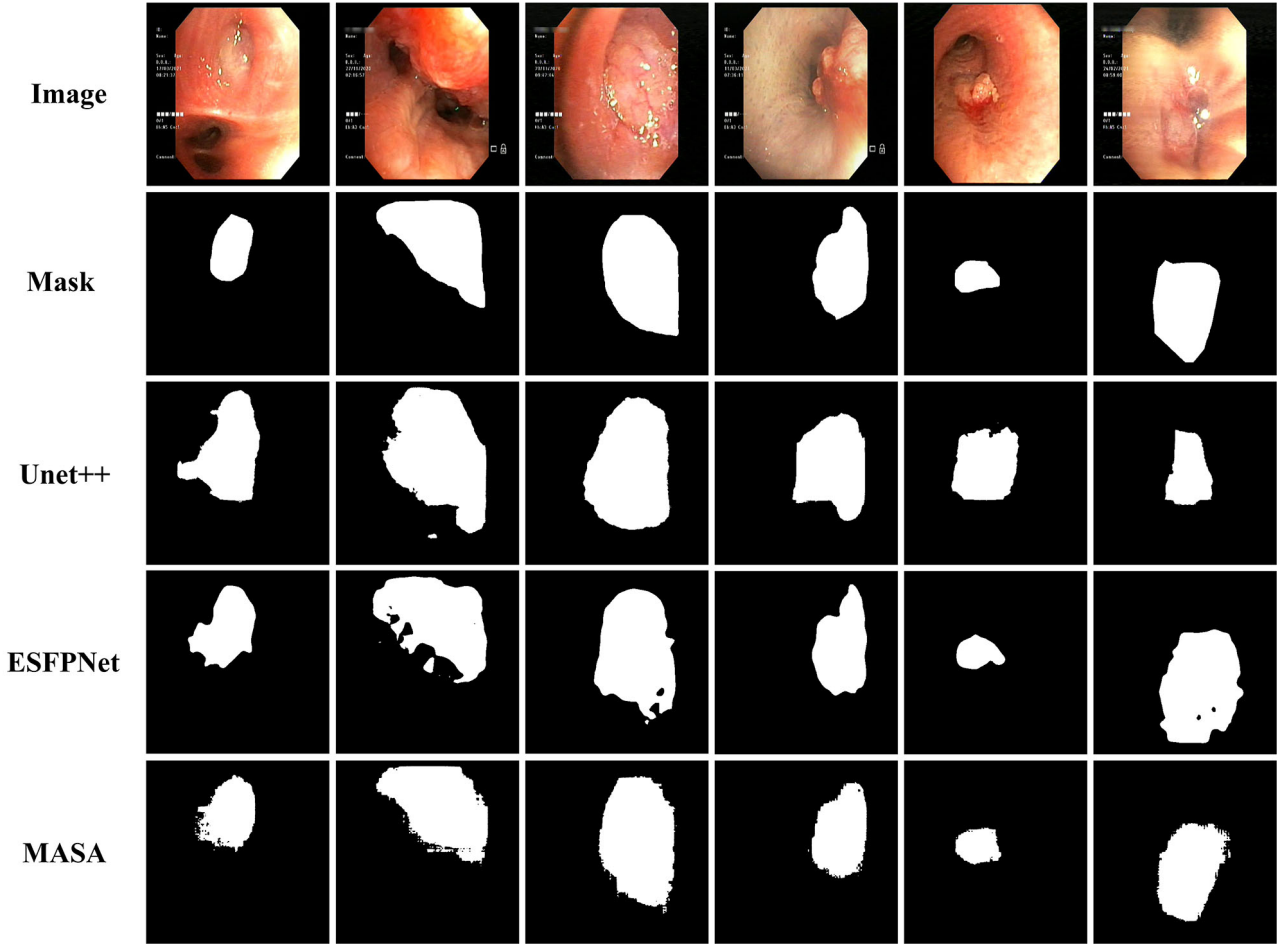
Segmentation qualitative result.

**Figure 7** presents qualitative segmentation outcomes for six cases with morphologically diverse lesions. The ground-truth masks display variable size, shape, and boundary irregularity. Across these cases, MASA generated contiguous masks that closely matched the reference in both extent and geometry. Boundaries adhered to mucosal edges with limited spillover into background, internal voids were largely suppressed, and small targets were preserved without fragmentation.

In comparison, U-Net++ often truncated peripheral regions or leaked beyond lesion margins, resulting in systematic oversegmentation or undersegmentation. ESFPNet frequently produced perforated interiors and scattered spurious regions, particularly near specular highlights and textured mucosa. Overall, MASA maintained coherent shapes with minimal false regions and fewer holes, yielding the closest visual agreement with the ground truth among the three methods. These observations are consistent with the quantitative advantages in **Table 4** and indicate robust delineation under varied appearance and illumination conditions.

In summary, the qualitative evidence indicates that MASA mitigated artifacts characteristic of the baselines. U-Net++ often exhibited boundary leakage and mask fragmentation, whereas ESFPNet frequently showed perforated interiors and scattered spurious regions; these issues were markedly reduced in MASA’s outputs. The visual consistency aligns with the quantitative results in **Table 4** and suggests strong potential for reliable decision support in bronchoscopic assessment.

### 4.7 Sensitivity study

We conducted a sensitivity analysis to investigate the impact of task-weighting coefficients on model performance. Specifically, we evaluated different values of the classification branch weight while fixing the segmentation loss weights as $\lambda_{\text{BCE}}=1.0$ and $\lambda_{\text{IoU}}=2.0$.

**Figure 8:**
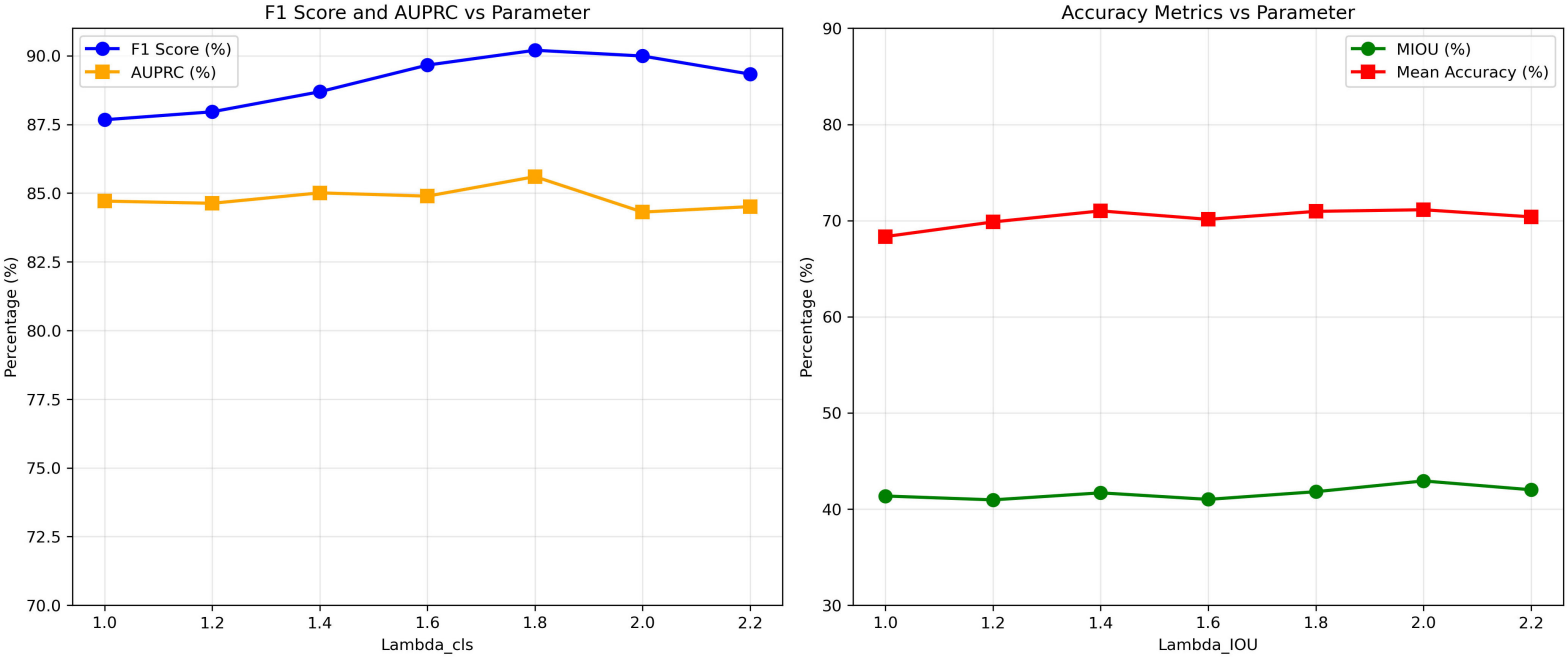
Sensitivity study on lambda.

As shown in **Figure 8**, the Macro-F1 (short for F1) score and AUPRC generally improve as the classification weight increases from 1.0 to 1.8, reaching the best performance at *λ*_cls_ = 1.8 (F1 = 90.20%, AUPRC = 85.60%). Further increasing the weight leads to a noticeable degradation, indicating that excessive emphasis on the classification branch adversely affects overall performance.

For segmentation, the mIoU and mAcc exhibit a gradual upward trend with increasing classification weight, peaking approximately $\lambda_{\text{cls}}=2.0$. However, performance gains beyond $\lambda_{\text{cls}}=1.8$ are marginal and accompanied by fluctuations, suggesting diminishing returns and potential task imbalance.

Based on these observations, we set the multi-task loss weights to $\lambda_{\text{seg}}=1.0$ and $\lambda_{\text{cls}}=1.8$, which provides a favorable trade-off between segmentation and classification performance. This choice ensures stable optimization while maintaining strong performance across both tasks.

### 4.8 Model complexity comparison

**Table 5** compares the model complexity of MASA against two representative baselines, U-Net++ (22) and ESFPNet (9), in terms of the number of trainable parameters (in millions) and computational cost (GFLOPs).

**Table 5:** Comparison of model parameters and GFLOPs.

Model	# Parameters (M)	FLOPs (G)	Inference time (ms/frame)
U-Net++	9.2	65.7	180
ESFPNet	61.7	23.9	37
MASA (ours)	239	40.6	46

U-Net++ has 9.2M parameters and requires 65.7 GFLOPs, reflecting its relatively lightweight design based on nested dense U-Net architectures. ESFPNet, which incorporates efficient transformer blocks and edge-aware feature enhancement, increases complexity moderately to 61.7M parameters and 23.9 GFLOPs, enabling real-time lesion segmentation in autofluorescence bronchoscopy.

Our proposed MASA model has 239M parameters and 40.6 GFLOPs. The higher complexity arises from its multi-adapter encoder (HOPE, PULSE, and PEAK) and the dual-task LUCID decoder, which jointly process spatial, frequency, and positional information for unified segmentation and classification. Despite the increased scale, MASA remains computationally tractable—trained efficiently on dual RTX 4090 GPUs with a batch size of 1—and achieves significant performance gains in both lesion delineation and malignancy diagnosis.

Thus, MASA introduces moderate computational overhead compared to ESFPNet but delivers substantial improvements in both segmentation fidelity and diagnostic reliability, making it well-suited for deployment in clinical bronchoscopy workflows where both accuracy and interpretability are paramount.

## 5 Conclusion

In this study, we developed the MASA, a unified encoder–decoder framework for joint lesion segmentation and lung cancer diagnosis in bronchoscopic images. The encoder integrates the HOPE, PULSE, and PEAK modules, enabling the joint fusion of spatial, frequency, and positional information for comprehensive feature representation. Coupled with the dual-task LUCID decoder, MASA addresses the limitations of conventional approaches that handle segmentation and classification separately, producing more consistent and clinically interpretable outputs. MASA operates directly on visual evidence of lesions, enabling spatially precise diagnosis. Furthermore, whereas approaches like CHASHNIt rely on *post-hoc* explainability that may lack fidelity to internal model reasoning, MASA provides intrinsic interpretability via its dual output: a clinically aligned segmentation mask and a classification decision derived from the same spatially grounded features. Its multi-adapter architecture—integrating spatial, frequency, and positional cues—enhances robustness without resorting to synthetic data. Thus, MASA balances architectural sophistication with clinical transparency, offering a diagnostically actionable and inherently explainable pipeline uniquely suited to real-time bronchoscopic decision support.

Although the LUCID decoder shares conceptual similarity with the SAM two-way transformer, direct comparison with SAM or MedSAM was not included because these models require user prompts and are not designed for the fully automatic bronchoscopy setting. Furthermore, MedSAM variants are tailored to CT or MRI and do not account for the illumination variability, mucosal deformation, and nonrigid geometry of airway imagery. MASA differs fundamentally by integrating multi-adapter encoding and a unified segmentation classification decoder, enabling prompt-free inference and superior lesion sensitivity. Future work will include SAM baselines using standardized synthetic prompts to provide a controlled comparison under the bronchoscopy domain.

Extensive experiments on the BM-BronchoLC dataset demonstrate MASA’s superior performance over established baselines. For lesion segmentation and classification, MASA outperforms the representative methods. Qualitative analysis further confirmed MASA’s ability to delineate subtle lesions and reduce diagnostic errors under challenging imaging conditions. With its unified and modular design, MASA not only streamlines the diagnostic workflow but also enhances interpretability in clinical practice. Future work will focus on prospective clinical validation, testing in diverse patient populations, and extending the framework to multi-class and multi-modal bronchoscopic analyses. Overall, MASA provides a robust and interpretable solution with strong potential to advance computer-aided bronchoscopy for early lung cancer detection.

In the future, we will focus on rigorous clinical validation of MASA in real-world bronchoscopy settings. This will include the design of well-defined retrospective and prospective clinical studies with clearly specified patient cohorts, standardized image acquisition protocols, and expert-driven annotation procedures. Comprehensive patient-wise evaluation will be conducted using clinically relevant metrics for both lesion segmentation and diagnostic classification. In addition, validation across multiple centers and diverse patient populations will be pursued to assess generalizability and robustness. Beyond binary diagnosis, future extensions will explore multi-class lesion characterization and multi-modal integration with complementary clinical information, further advancing the applicability of MASA for computer-aided bronchoscopy and real-time clinical decision support.

## Data Availability

The original contributions presented in the study are included in the article/supplementary material. Further inquiries can be directed to the corresponding authors.
